# Advances in the identification of novel cell signatures in benign prostatic hyperplasia and prostate cancer using single-cell RNA sequencing

**DOI:** 10.3389/fimmu.2025.1684895

**Published:** 2026-01-09

**Authors:** Yu Pan, Qingqing Song, Bingjie Lai, He Ma

**Affiliations:** 1 Department of Urology, The Second Hospital of Jilin University, Changchun, China; 2 Department of Obstetrics and Gynecology, The Second Hospital of Jilin University, Changchun, China; 3 Department of Intensive Care Unit, The Second Hospital of Jilin University, Changchun, China; 4 Department of Anesthesiology, The Second Hospital of Jilin University, Changchun, China

**Keywords:** benign prostatic hyperplasia, prostate cancer, chronic prostatic diseases, cell subpopulations, molecular signatures, single-cell RNA sequencing

## Abstract

Nowadays, chronic benign and malignant prostatic diseases are prevalent, costly, and impose a significant burden. Benign prostatic hyperplasia (BPH), a common condition in the aging population, often coexists with localized prostate cancer (PCa). These diseases likely share underlying molecular mechanisms, which remain poorly understood. The exploration of novel cell subpopulations and specific biomarkers for accurate diagnosis and treatment of prostatic diseases is ongoing and holds great clinical promise. Prostate cell proliferation and immune inflammation are key contributors to the progression of BPH and PCa, involving various prostate and immune cell subpopulations. This raises important questions about how specific cell types drive phenotypic heterogeneity. Advanced single-cell RNA sequencing (scRNA-seq), a cutting-edge technology, offers unparalleled insights at the single-cell level. Similar to a microscope that identifies cell types within tissue samples, scRNA-seq elucidates cellular heterogeneity and diversity within single cell populations, positioning itself as a future-leading sequencing technology. Considering that BPH and PCa share androgen-dependent growth, chronic inflammation and specific microenvironmental changes, this review discusses recent discoveries of novel cell subpopulations and molecular signatures in BPH and PCa that can be dissected by scRNA-seq. It aims to help researchers better understand the molecular pathogenesis of these conditions while offering new therapeutic possibilities for clinical management of benign and malignant prostatic disorders.

## Introduction

Benign and malignant prostatic diseases, such as benign prostatic hyperplasia (BPH) and prostate cancer (PCa), are prevalent among middle-aged and elderly men worldwide and continue to impose significant health and economic burdens (1, 2). BPH is a condition that increases in prevalence with age. It is primarily characterized by the enlargement of the prostate and surrounding tissues, which often results in lower urinary tract symptoms (LUTS) and significantly affects quality of life (3, 4). Lifestyle modifications and pharmacotherapy are currently the primary treatments for patients. However, pharmacotherapy is associated with notable side effects, including orthostatic hypotension, dizziness, asthenia, fatigue, headache, erectile dysfunction, and ejaculatory dysfunction (5, 6). PCa, the most common malignant tumor affecting the male genitourinary system, remains a significant health threat to elderly men (7, 8). In the progression of PCa, prostatic intraepithelial neoplasia represents the initial stage of malignant transformation, followed by localized PCa, advanced locally invasive adenocarcinoma, and ultimately metastatic PCa (9). Unfortunately, approximately 10%-15% of patients receive a PCa diagnosis after metastasis has occurred (10). The majority of metastatic PCa patients develop bone metastases, which significantly worsen their prognosis (11, 12). Recent studies have identified secondary colorectal cancer in PCa patients as an independent risk factor influencing prognosis (13, 14). In general, patients with secondary cancer exhibit significantly lower survival rates. Therefore, understanding how PCa progresses with or without secondary cancer and identifying its molecular signatures are crucial for achieving accurate diagnosis and early treatment of these patients.

Although BPH and PCa are distinct diseases, they share several significant characteristics. Both conditions are highly prevalent among aging males. PCa is the second most common cancer in men, while BPH affects approximately 70% of men by the age of 70. Although the relationship between BPH and PCa remains controversial and is influenced by many issues such as detection bias, shared risk factors, and different study designs, there’s a debate that BPH increases the risk of developing PCa by 2- to 12-fold, highlighting the importance of comprehensive clinical guidelines and effective risk stratification methods (15). Regarding molecular mechanisms, both diseases are linked to inflammation, oxidative stress, and fibrosis. Growing evidence suggests that chronic inflammation is a critical link between BPH and PCa, with interleukin (IL)-6, IL-8, IL-15, and IL-17 playing roles in the progression of both diseases (16, 17). Androgens are essential growth factors for normal prostate development and also drive the progression of BPH and PCa (18). Consequently, androgen deprivation therapy is a treatment option for both conditions (19). Prostate epithelial cells, a key cell type in the prostate, play a crucial role in maintaining tissue integrity. Malignant transformation of these cells in BPH or PCa underscores their importance as pivotal therapeutic targets (20). Beyond epithelial cells, the prostatic stroma also plays a critical role in the two diseases, involving sex steroid hormone receptors like the androgen receptor (AR) and estrogen receptor-α (21). Thus, BPH and PCa exhibit common pathological features. Moreover, an association exists between BPH and PCa, suggesting that treatments for BPH should also possess chemopreventive effects against PCa. However, the nature of whether this relationship is causal or coincidental remains a subject of ongoing research and debate.

However, the current understanding of two diseases has long been constrained by limitations in research technology. Recently, advances in sequencing technology and omics studies have accelerated research on BPH and PCa, positioning these innovations as critical driving forces in the field (22–24). Single-cell RNA sequencing (scRNA-seq) is a powerful tool for revealing the complex mechanisms underlying diseases. It allows for the investigation of gene expression at the individual cell level, offering insights into BPH and PCa, particularly regarding their plasticity, metastatic potential, and tumor microenvironment (TME) (25, 26). This review summarizes the latest findings on novel cell subpopulations and molecular signatures identified in BPH and PCa through scRNA-seq. It aims to highlight the molecular pathogenesis of two different disease states and provide new avenues for the clinical management of benign and malignant prostatic disorders. The following chapters will provide a detailed summary and discussion of new cell subpopulations and molecular signatures identified in BPH and PCa through scRNA-seq.

## Resolving cellular heterogeneity: the power of single-cell RNA sequencing

So far, scRNA-seq has firmly established itself as a transformative technology in biomedical research, moving beyond bulk tissue analysis to provide an unprecedented, high-resolution view of cellular heterogeneity and function. By capturing the gene expression profile of individual cells, it has revolutionized our understanding of basic biology, revealing previously unappreciated cellular diversity within tissues, identifying rare cell populations, and uncovering complex cellular interactions and developmental trajectories (27, 28). This granular view is crucial, as traditional methods that average signals across thousands of cells often mask critical differences that drive health and disease. The technology’s applications now span a vast spectrum, from delineating the intricate cellular architecture of organs and tracking cell fate decisions during development to characterizing the complex ecosystem of the tumor microenvironment and deciphering the nuanced responses of immune cells to pathogens (29, 30).

In clinical research, scRNA-seq is rapidly transitioning from a discovery tool to an engine for diagnostic, prognostic, and therapeutic innovation, particularly in oncology (31). By analyzing cancers at single-cell resolution, researchers can identify specific cell populations that drive disease aggressiveness and therapeutic resistance, such as cancer stem cells (CSC). For instance, in hepatocellular carcinoma, scRNA-seq analysis has identified a diagnostic model based on 4 CSC-related genes that effectively stratifies patients into high- and low-risk groups, directly informing prognosis and potential treatment strategies (32). Similarly, in pancreatic adenocarcinoma, integrated transcriptome and scRNA-seq analysis have revealed the prognostic significance of the GBP4 gene. Researchers have demonstrated that GBP4 promotes cancer cell proliferation and invasion, and developed a 5-gene prognostic signature that correlates with distinct immune infiltration patterns, offering new avenues for patient stratification and targeted therapy (33). Beyond cancer, scRNA-seq is pinpointing novel therapeutic targets in other complex diseases, such as identifying JUNB/SPI1-driven inflammatory programs and novel communication axes in myeloid cells of sepsis (34).

The current state of this field is marked by technological and analytical maturation that is pushing the boundaries of what is possible. The integration of scRNA-seq with other single-cell modalities, such as single-cell assay for transposase-accessible chromatin using sequencing (scATAC-seq) and spatial transcriptomics (ST), is a powerful trend (35). This multi-omics approach provides a more holistic view, linking gene expression to epigenetic regulation and cellular location within a tissue. Furthermore, the field is increasingly relying on sophisticated computational biology and machine learning to manage the scale and complexity of single-cell data, transforming it into clinically actionable knowledge, such as the prognostic models now being developed for various cancers (36).

## Potential value of single-cell RNA sequencing in studying benign prostatic hyperplasia and prostate cancer

The progression of BPH and PCa is driven by a range of critical pathological processes. These include prostate inflammation, oxidative stress, fibrosis, and the interaction between tumor cells and the TME (37, 38). Identifying various subpopulations of epithelial cells, stromal cells, and immune cells, as well as their distinct molecular signatures, is crucial for understanding the complex mechanisms underlying chronic prostatic diseases. The advent of scRNA-seq has revolutionized the study of BPH and PCa. This technology addresses the limitations of traditional methods, such as fluorescence-activated cell sorting, which struggle to elucidate cell-specific gene expression. As a result, scRNA-seq shows great potential for revealing the heterogeneity and functional changes associated with BPH and PCa (39). When combined with Kyoto Encyclopedia of Genes and Genomes (KEGG) pathway analysis, Gene Ontology (GO) analysis, and cell trajectory analysis, scRNA-seq offers new insights into cellular functions, predicts signaling pathways, and illuminates processes of cell differentiation and development (40, 41).

Notably, scRNA-seq not only reveals the landscape and functions of normal prostate cells, stromal cells, and immune cells, but also identifies new populations of these cells that may be implicated in the onset and progression of BPH and PCa, as shown in **Tables 1**, **2**. For instance, Henry et al. have characterized three types of prostate epithelial cells—luminal epithelial cells, basal epithelial cells, and neuroendocrine cells—as well as two novel types—hillock cells and club cells—which are classified as either malignant or non-malignant. Of the two novel epithelial cell populations, one consists of KRT5/KRT14/KRT13-positive hillock cells, which are localized in the central zone (CZ) around the prostatic urethra, collecting ducts, and ejaculatory ducts. The other is composed of KRT5/KRT8/SCGB1A1-positive club cells, which are found in the prostatic urethra and collecting ducts (42). Therefore, scRNA-seq uncovers epithelial cell heterogeneity and the pathological characteristics of chronic benign and malignant prostatic diseases, offering a solid molecular foundation for targeted treatments of BPH and PCa, and holding promise for enabling personalized therapeutic approaches.

**Table 1:** Identification of novel cell subpopulations in benign prostatic hyperplasia using single-cell sequencing.

Cell types	Specific markers of subpopulations	Species	Specimens	Single-cell seq techniques	Effects	Clinical relevance	Ref.
Epithelial cells							
BE cells	c-Fos	Human	Prostate tissues	10× Genomics	BE5 cell subgroup in patients with BPH was its heightened hypoxia and upregulated FOS.	Mechanistic insight into disease	(51)
Prostatic luminal epithelial cells	ESR1	Mouse	Fresh prostate tissues	10× Genomics	Prostatic Esr1+ luminal epithelial cells were enhanced in the absence of SRD5A2.	Mechanistic insight into therapy resistance	(52)
Prostate epithelial cells	NRK	Human	Prostate tissues	Data reanalysis (GSE172357)	Upregulated NRK was associated with prostatic stromal cell proliferation, apoptosis, cell cycle, migration, fibrosis and EMT process.	Novel therapeutic targets	(54)
Prostate epithelial cells	NOTCH1	Human	Prostate tissues	Data reanalysis (GSE145928)	Over-activated Notch1 signaling induces prostatic enlargement.	Mechanistic insight into disease	(53)
Immune cells							
Lipid-rich macrophages	TREM2, MARCO	Human	Prostate transition zone tissues	10× Genomics	Lipid-rich macrophages may exacerbate LUTS in patients with large prostates.	Novel therapeutic targets	(61)
CXCL17-mediated macrophages	CXCL17	Mouse	The prostate lumen	Paired-end sequencing	CXCL17-mediated macrophage migration is a critical initiating event in BPH.	Mechanistic insight into disease	(62)

BE, basal epithelial; CXCL17, C-X-C motif chemokine 17; EMT, epithelial-mesenchymal transition; ESR1, estrogen receptors alpha; LUTS, lower urinary tract symptoms; MARCO, macrophage receptor with collagenous structure; NRK, Nik related kinase; TREM2, triggering receptor expressed on myeloid cells 2.

**Table 2:** Identification of novel cell subpopulations in prostate cancer using single-cell sequencing.

Cell types	Specific markers of subpopulations	Species	Specimens	Single-cell seq techniques	Effects	Clinical relevance	Ref.
Cancer cells							
PCa cells	COX-2, prolactin, prolactin receptor	Mouse	Primary tumor, lung metastases	SMART-Seq	COX-2 mediates tumor-stromal prolactin signaling to initiate tumorigenesis.	Diagnostic biomarker & therapeutic target	(78)
Double-negative PCa populations	KRT7; SOX2, FOXA2	Human; mouse	TRAMP tumor tissues	10× Genomics	Novel double-negative PCa subtypes were unveiled.	Mechanistic insight into disease	(79)
Four transcriptional subtypes of PCa cells	ZEB1, KLK3, TACSTD2, KRT15	Human	PCa samples	10× Genomics	PCa cells were stratified into four transcriptional subtypes, EMT-like, luminal A-like, luminal B/C-like, and basal-like.	Prognostic biomarker	(80)
PCa cells	SMA/FOLH1, B7-H3/CD276	Human	PCa samples	10× Genomics	ICC/IDC cancer cells express genes associated with metastasis and targets with potential for therapeutic intervention.	Prognostic biomarker	(91)
Plasticity-related cancer cells	HMMR	Human	PCa samples	Data reanalysis (GSE230282)	A novel lineage plasticity-related cancer cell type was involved in PCa progression.	Prognostic biomarker	(83)
PCa cells	NEAT1	Human	Prostate biopsies	Data reanalysis (GSE176031)	NEAT1 was a potential biomarker and therapeutic target of PCa.	Diagnostic biomarker & therapeutic target	(84)
Epithelial cells							
PCa-associated epithelial cells	AR	Human	Prostate biopsies	Drop-seq	Epithelial cells from PCa tissues were generally upregulated in AR signaling.	Mechanistic insight into disease	(98)
Basal/intermediate epithelial cells	PIGR, MMP7, AGR2	Human	PNI-PCa samples	10× Genomics	Basal/intermediate cells communicate closely with neural cells played a potential role in PNI-PCa progression.	Mechanistic insight into disease	(99)
Intermediate luminal epithelial cells	AR	Mouse	Ventral prostate lobes	10× Genomics	Distinct epithelial populations are exquisitely sensitive to translation inhibition.	Mechanistic insight into disease	(100)
Luminal epithelial cells	HPN	Human	PCa samples	10× Genomics	A distinct subgroup of luminal cells critical for PCa diagnosis and stratification was identified along with its marker gene HPN.	Diagnostic biomarker & therapeutic target	(101)
Club-like epithelial cells	CTSB, MMP10, PLAU, IGFBP3, ICAM1	Human	PCa samples	Data reanalysis (GSE278936)	Club-like epithelial cells act as an interface between the prostate and the immune system.	Mechanistic insight into therapy resistance	(103)
PCa-associated epithelial cells	TMED3	Human	PCa samples	Data reanalysis (GSE176031)	TMED3 promotes malignant proliferation of PCa cells	Prognostic evaluation & immune classification of PCa	(104)
Stromal cells							
Stromal cells	COX-2, prolactin, prolactin receptor	Mouse	Primary tumor, lung metastases	SMART-Seq	COX-2 mediates tumor-stromal prolactin signaling to initiate tumorigenesis.	Diagnostic biomarker & therapeutic target	(78)
Fibroblasts, smooth muscle cells	APOD, PTGDS, PTGS2, MMP2	Human; mouse	Fresh PCa samples	10X Genomics	A receptor-ligand interaction analysis revealed a central role for fibroblasts in PCa.	Mechanistic insight into disease	(115)
Immune cells							
Macrophages	NCF4	Human	Primary PCa samples	Data reanalysis (GSE141445)	The NCF4 gene has been identified as the hub gene in MRS.	Mechanistic insight into disease	(129)
	PPIF	Human	Primary PCa samples	Data reanalysis (GSE176031)	PPIF was significantly higher in TAMs from tumor tissue than normal tissue,	Therapeutic target	(130)
	ATF3	Human	PRAD samples	Data reanalysis (GSE153892)	Abnormal ATF3 may be related to docetaxel and cabazitaxel resistance in PCa cells.	Patient stratification& predicting treatment response	(132)
	M1 and M2 markers	Human	Prostate biopsies	SORT-seq	Five macrophage clusters were identified, suggesting the role of TAMs in PCa.	Prognostic evaluation	(131)
	MAC	Human	Primary PCa samples	10× Genomics	MAC-MT contributes to tissue zinc accumulation in homeostasis but shows elevated inflammation in tumors.	Prognostic biomarker	(133)
T cells	KLK3	Human	Fresh PCa samples	10× Genomics	Ectopic expression of KLK3 in T cells is associated with micrometastases.	Mechanistic insight into disease	(137)
	EP4	Human	Fresh PCa samples	10X Genomics	EP4 serves as a target for restoring T-cell infiltration and sensitizing PCa to immunotherapy.	Novel therapeutic target	(138)

AR, androgen receptor; ATF3, activating transcription factor; COX-2, cyclooxygenase-2; CTSB, TME-altering proteases cathepsin β; EMT, epithelial-mesenchymal transition; EP4, E2 receptor 4; HMMR, hyaluronan mediated motility receptor; HPN, hepsin; ICAM1, intercellular adhesion molecule 1; IGFBP3, insulin-like growth factor-binding protein 3; KLK3, kallikrein-related peptidase 3; MAC, metallothionein; MMP, matrix metalloproteinases; PLAU, urokinase; PPIF, peptidylprolyl isomerase F; PRAD, prostate adenocarcinoma; TAMs, tumor-associated macrophages; TMED3, transmembrane p24 trafficking protein 3; NCF4, neutrophil cytosolic factor 4.

## An update on the identification of novel cell subpopulations in benign prostatic hyperplasia using single-cell RNA sequencing

BPH is a highly prevalent condition, affecting approximately 25% of men over their lifetime. It is commonly managed using α-adrenergic receptor antagonists (α-blockers) or 5α-reductase inhibitors (43, 44). BPH is characterized by excessive proliferation of prostatic epithelial and stromal cells, which results in prostate enlargement and LUTS (45). Additional key factors contributing to BPH include inflammation, oxidative stress, and increased immune cell infiltration. The process involves macrophages, along with the accumulation of inflammatory mediators and growth factors in the tissue environment (46, 47). Although several drugs are available for treating BPH, their long-term effects—whether used alone or in combination—on the risk of clinical progression remain uncertain. The Medical Therapy of Prostate Symptoms (MTOPS) trial, the largest clinical study on BPH, has tracked 3,047 men over five years. It revealed that finasteride reduced the progression of LUTS by only 34% in BPH patients (48). Numerous side effects of current BPH medications have spurred growing interest in identifying novel therapeutic targets.

Therefore, elucidating the complex interactions between hormonal and signaling pathways using scRNA-seq is essential for advancing research into more effective treatments for prostatic diseases. This research involves the studies of various cell populations, including epithelial, stromal, and immune cells. Detailed descriptions of these findings are provided in the following paragraphs and **Table 1**.

### Epithelial cells

Roles of androgen response, hypoxia, and epithelial-mesenchymal transition (EMT) in the prostatic epithelium have been recognized as contributors to BPH, underscoring the essential function of epithelial cells in proliferative nodular formation (49, 50). However, technological limitations currently impede *in vivo* investigations in patients with BPH. Specific alterations and functional changes in epithelial cells during proliferative nodular formation are not yet fully understood. Fei and colleagues have revealed a comprehensive *in vivo* landscape of epithelial cells during nodular formation in BPH patients, identifying one fibroblast cluster and four distinct epithelial clusters. Of four epithelial clusters, one is predominantly localized within the nodule, while the other three are primarily distributed in extra-nodular regions. The BE5 cell subgroup in BPH patients is distinguished by increased hypoxia and elevated FOS expression. These findings provide novel insights into the initiation and progression of BPH, offering potential therapeutic implications (51). Sharkey et al. have utilized a *Srd5a2^-/-^* mouse model and scRNA-seq to investigate effects of SRD5A2 deficiency on prostate cellular heterogeneity. Significant changes in luminal epithelial cell (LE) populations are observed, including an increased proportion and enhanced proliferative phenotype of estrogen receptor 1 (ESR1)+ LE2 cells, driven by an SRD5A2-independent ESR1 differentiation trajectory (52).

Beyond original research, some scientists have reanalyzed raw single-cell data retrieved from the public Gene Expression Omnibus (GEO) database (53, 54). He and colleagues have demonstrated that silencing Nik-related kinase (NRK) inhibits stromal cell proliferation, migration, fibrosis, and the EMT process, while promoting apoptosis and inducing cell cycle arrest. Conversely, NRK overexpression in prostate epithelial cells yields opposing effects. Additionally, NRK knockdown reverses induced fibrosis and the EMT process. Moreover, NRK expression levels positively correlate with α-SMA, collagen-I, and N-cadherin, while negatively correlating with E-cadherin expression (54). Kang et al. have reanalyzed scRNA-seq data from prostates of patients with and without BPH, and revealed that overactivated Notch1 signaling induces prostatic enlargement by enhancing AR sensitivity, disrupting mitochondrial metabolism, increasing ROS expression, and expanding progenitor cell populations. These abnormalities are effectively reversed by antioxidant N-acetyl-L-cysteine (NAC) treatment (53). In brief, recent scRNA-seq analyses have revealed that epithelial cell heterogeneity and dysfunction are central to the pathogenesis of BPH.

### Stromal cells

Similar to epithelial cells, prostate stromal cell proliferation is a characteristic feature of BPH (55, 56). Stromal and epithelial components contribute to ~80% and ~20% of the hyperplastic volume of the prostate gland, respectively. Notably, stromal cells show significantly higher proliferation levels than epithelial cells, highlighting that BPH is characterized by stromal-dominant growth (57, 58). Therefore, elucidating the molecular pathways governing stromal cell proliferation and programmed cell death in the prostate is crucial for developing novel therapies. However, no original studies have specifically focused on stromal cells in the context of BPH, particularly using scRNA-seq. This underscores the urgent need for further research in this area. Collectively, despite being the dominant driver of BPH progression, prostate stromal cells remain significantly understudied at the single-cell level.

### Immune cells

Previous research has explored the association between immune cells and BPH. However, it remains unclear whether specific immune subpopulations are disproportionately represented in BPH tissues, warranting further investigation (59, 60). Lanman and colleagues have hypothesized that changes in macrophage phenotypes associated with increasing prostate size may involve metabolic alterations, leading to epithelial or stromal hyperplasia in the prostate. The abundance of *TREM2^+^* and *MARCO^+^* macrophages is positively correlated with patient body mass index (BMI) and urinary symptom scores. *TREM2^+^* macrophages from BPH tissues exhibit significantly higher levels of neutral lipids compared to *TREM2^-^* macrophages. These findings identify two novel immune subpopulations in BPH, *TREM2^+^* and *MARCO^+^* macrophages, and suggest that lipid-rich macrophages may contribute to the worsening of LUTS in patients with enlarged prostates (61). Silver et al. have identified the upregulation of epithelial-derived CXCL17, a monocyte attractant, in T + E2 prostates. This suggests its role in increasing macrophage numbers and promoting their migration to the lumen (62). In summary, emerging evidence implicates specific immune subpopulations, particularly lipid-rich macrophages, in directly contributing to BPH pathogenesis and symptom severity.

## An update on novel cell subpopulations in prostate cancer identified using single-cell sequencing

PCa is the most common tumor among men in Western nations, with its incidence increasing in recent years. Radical prostatectomy (RP) remains the most commonly used standard treatment for clinically localized PCa (63). However, many patients unfortunately progress to metastatic PCa even after successful RP therapy (64, 65). PCa is challenging to detect at an early stage due to a lack of symptoms. Thus, early detection of disease progression is crucial for reducing PCa-related mortality (66, 67). PCa can be detected using methods such as measuring plasma levels of prostate-specific antigen (PSA) and performing rectal examinations, with biopsy required to confirm the diagnosis (68, 69). Advanced technologies, as exemplified by scRNA-seq, have revolutionized our understanding in the pathological process of PCa (22, 25, 39). Notably, scRNA-seq enables characterization of diverse subpopulations, including cancer cells, cancer-associated epithelial cells, stromal cells, and infiltrating immune cells in PCa, offering valuable insights into disease mechanisms. Building on this rationale, this review summarizes recent findings on novel cell subpopulations in PCa identified via scRNA-seq, highlighting cell-specific markers that may serve as potential therapeutic targets (**Table 2**) to enhance precise diagnosis and treatment.

### Prostate cancer cells

Similar to healthy prostate cells, PCa cells rely on androgens for survival and proliferation, particularly during the initial stages of PCa (70, 71). PCa cells typically exist in two distinct states: an epithelial state, marked by high polarity, strong cell-cell adhesion, cobblestone morphology, and low chemoresistance; and a mesenchymal state, characterized by low polarity, weak cell-cell adhesion, elongated spindle-like morphology, high migratory potential, and elevated chemoresistance (72, 73). Notably, the EMT, a biochemical process where cells lose epithelial features and acquire mesenchymal traits, is associated with chemoresistance and metastasis, presenting significant challenges in PCa treatment (74–76). ScRNA-seq offers a novel approach to longitudinally examine PCa progression by investigating the lineage and evolution of tumor cells. Cell trajectory analysis can reveal hidden subtypes that potentially play a key role in tumor progression (77). This method provides deeper insights into cellular dynamics and tumor heterogeneity, enabling a more comprehensive understanding of cancer progression (22, 25, 39).

Using scRNA-seq in immune-compromised mouse xenograft models and *in vitro* reconstitution experiments, Zheng and partners have identified a distinct tumor cell subtype derived from PCa micro-metastases in mice. This subtype exhibits significant differences from primary site tumor cells, with high expression of the prolactin receptor (PRLR). Analysis of multiple human cancers reveals distinct expression patterns of cyclooxygenase-2 (COX-2), prolactin, and PRLR in tumor and stromal cells. Subsequent experiments uncover a key intercellular pathway: elevated COX-2 levels in tumor cells promoted prostaglandin E2 (PGE2 synthesis, which upregulates nuclear receptor 4A (NR4A) in stromal cells, stimulating prolactin secretion (78). The discovery of this novel tumor cell subtype highlights the critical role of COX-2 in PCa progression. In addition, Cheng et al. have identified two novel double-negative PCa subtypes in patient samples using scRNA-seq: KRT7 cells with elevated KRT7 expression and progenitor-like cells expressing SOX2 and FOXA2. These subtypes are distinct from NEPCa and exhibit stem/progenitor traits (79). Notably, they have compiled scRNA-seq data from multiple studies to create the Human Prostate Single Cell Atlas (HuPSA) and the Mouse Prostate Single Cell Atlas (MoPSA). Ge and co-authors have conducted scRNA-seq on samples from 14 untreated PCa patients with the identification of four transcriptional subtypes, including EMT-like, luminal A-like, luminal B/C-like, and basal-like. These subtypes are hierarchically organized into stem cell-like and differentiated states. Notably, multiple subclones within a single primary tumor exhibits distinct combinations of preferential subtypes. Furthermore, subclones show varying communication strengths with other cell types in the tumor ecosystem, potentially influencing their distinct transcriptional subtypes (80).

Lineage plasticity is a well-established factor contributing to therapy resistance and trans-differentiation into neuroendocrine lineages in PCa (81, 82). To investigate this, Zhao et al. have re-analyzed a comprehensive scRNA-seq meta-atlas, identifying a novel population of lineage-plasticity-related cells characterized by low AR activity, stemness-like traits, and high expression of hyaluronan-mediated motility receptor (HMMR), which may contribute to poor prognosis in PCa (83). Furthermore, they have identified HMMR as a key cell surface marker for lineage plasticity-related cells (LPCs), which is associated with aggressive tumor progression and poor prognosis in PCa. Notably, HMMR expression is transcriptionally repressed by AR and plays a pivotal role in neuroendocrine differentiation and the aggressive phenotype of advanced PCa cells (83). Li et al. have employed scRNA-seq to cluster PCa, BPH, and normal prostate samples, identifying specific cellular subsets associated with PCa progression. They have identified 10 cellular subsets, observing variations in their proportions among normal, BPH, and tumor groups. Significant differences are noted between basal intermediate and luminal epithelial cells during the transition from prostate hyperplasia to cancer. Interestingly, NEAT1 expression is elevated in PCa, highlighting its potential as a biomarker and therapeutic target for the disease (84).

In addition to cancer cells, researchers have investigated concurrent changes and interactions among other cell types, such as epithelial and immune cells, within the TME (85, 86). Both immune cells and the TME play critical roles in the development, progression, and metastasis of PCa (87, 88). The interplay between the immune system and the PCa microenvironment is dynamic, with immune cells exhibiting both inhibitory and promotive effects on cancer growth (89, 90). For example, Wong et al. have identified cell-intrinsic and tumor microenvironmental pathways underlying aggressive disease using single-cell analysis of cribriform PCa. The cribriform PCa TME is characterized by enhanced angiogenesis, immunosuppressive fibroblasts (*CTHRC1^+^ASPN^+^FAP^+^ENG^+^*), reduced T-cell numbers, increased T-cell dysfunction, and elevated *C1QB^+^TREM2^+^APOE^+^* M2 macrophages. These findings indicate that cancer cell-intrinsic pathways and a complex immunosuppressive TME drive the aggressive phenotype of cribriform PCa (91). Beyond T-cell alterations, the TME of cribriform PCa exhibits increased expression of CD163 and MSR1, markers of pro-tumor M2 macrophages. *CD163^+^* M2 macrophages are associated with worse clinicopathologic features and outcomes in PCa (92). Understanding the molecular-level interactions between the TME and PCa may inform the development of novel therapeutic strategies. Notably, additional scRNA-seq studies have explored the specific roles of various T-cell and macrophage subtypes in PCa, summarized in **Table 2** and discussed further in subsequent sections. Collectively, scRNA-seq has unveiled critical prostate cancer cell subtypes and their dynamic interactions within the TME, driving progression and therapy resistance.

### Epithelial cells

The development of cancer in prostate tissue involves complex interactions between tumor cells and surrounding epithelial and stromal cells. This process may occur multifocally, indicating that prostate epithelial cells undergo transitions in cellular states leading to carcinogenesis (93, 94). In recent years, several studies on molecular changes in PCa have primarily focused on unsorted bulk tissue samples, leaving a gap in our understanding of the adjacent epithelial cell states. Thus, PCa-associated epithelial cell states revealed by scRNA-seq have become a focal point of study (95–97).

It is well acknowledged that PCa-associated epithelial cells are commonly classified into basal epithelial cells, luminal epithelial cells, neuroendocrine cells, hillock cells, and club cells (42). Song et al. have identified heterogeneous cellular states in prostate epithelial cells characterized by high androgen signaling, which are enriched in PCa. They have also identified a population of tumor-associated club cells potentially linked to prostate carcinogenesis. ERG-negative tumor cells, compared to ERG-positive cells, exhibit shared heterogeneity with surrounding luminal epithelial cells and seem to generate common TME responses. Furthermore, integrated analysis of epithelial cells reveals upregulated AR signaling in PCa samples, potentially contributing to a more precise and thorough classification of cells during prostate carcinogenesis (98). Zhang and colleagues have performed scRNA-seq on tumor tissues from PNI-PCa patients, identifying 12 cell types, including two types of epithelial clusters and neural clusters. They have found that basal/intermediate cell subtypes, which show higher expression of PIGR, MMP7, and AGR2, play a crucial role in PNI-PCa progression. These findings enhance our understanding of PNI-PCa cellular heterogeneity and characterize the potential role of basal/intermediate cells in PNI-PCa development (99).

Germanos and coworkers have demonstrated that *Pten* deletion in mouse prostate epithelial cells leads to the generation of an intermediate luminal subtype, phenotypically similar to, but distinct from, the *Psca^+^/Krt4^+^/Tacstd2^+^* luminal progenitor populations in the WT prostate. They have observed that intermediate cells undergo significant changes in cell state upon castration, increasing their heterogeneity. This heterogeneity is marked by a spectrum of AR signaling, inversely correlated with both proliferation and translation activity. These results support the notion that basal cells can transdifferentiate into intermediate epithelial cells following *Pten* loss (100). Ma et al. have conducted scRNA-seq on PCa tissues from two patients and identified 15 clusters, including three distinct types of luminal clusters. Interestingly, they have identified an intriguing distinct subgroup of luminal cells, marked by the gene Hepsin (HPN), which is crucial for PCa diagnosis and stratification (101). Notably, this finding aligns with an earlier result from Goel et al., which suggests that HPN should be considered a novel immunohistochemical marker for the histopathological diagnosis of PCa based on immunostaining intensity in normal prostate, low-scoring, and high-scoring PCa (102).

Kiviaho et al. have identified club-like cells as a key epithelial cell subtype that serves as an interface between the prostate and the immune system. Tissue areas enriched with club-like cells exhibit depleted androgen signaling and upregulated expression of luminal progenitor cell markers. Club-like cells display a senescence-associated secretory phenotype, and their presence is linked to increased activity of polymorphonuclear myeloid-derived suppressor cells (PMN-MDSCs). These results suggest that club-like cells are associated with myeloid inflammation, which has previously been linked to resistance to androgen deprivation therapy. This finding provides a rationale for targeting these cells therapeutically (103). Zhu et al. have re-analyzed scRNA-seq data from previous studies and identified a comprehensive set of 543 epithelial cell marker genes (ECMGs). They have established a strong correlation between ECMGs and both the prognostic evaluation and immune classification of PCa. Notably, the ECMG prognostic signature (ECMGPS) demonstrates a strong predictive capability, surpassing traditional clinical features and 80 published signatures in both independence and accuracy across five cohorts. Furthermore, the role of TMED3 in promoting the malignant proliferation of PCa cells has been validated. These findings together highlight ECMGPS as a powerful tool for improving outcomes in PCa patients and provide a robust conceptual framework for the in-depth examination of PCa complexities (104). In short, scRNA-seq analyses have defined distinct, clinically relevant epithelial cell states that contribute to PCa initiation, progression, and therapy response.

### Stromal cells

Stromal cells, such as fibroblasts, smooth muscle cells, and vasculature-associated cells, make up a crucial component of the TME of PCa (105, 106). However, compared to epithelial cells, stromal cells have been largely overlooked. Recent studies have shown that the prostate stroma possesses distinct trophic and inductive properties, influencing prostate morphogenesis, specification, hormonal regulation, and pathological manifestations such as BPH and cancer (107, 108). Reactive stroma begins during the early stages of PCa development and coevolves with disease progression (109, 110). During this process, several key regulatory factors have been identified, including transforming growth factor β, IL-8, fibroblast growth factors, connective tissue growth factor, wingless homologs (Wnts), and stromal cell-derived factor-1 (107). Therefore, the stromal microenvironment has become a key factor in the growth and progression of PCa.

As previously discussed, Zheng and colleagues have employed single-cell transcriptome analyses to uncover a tumor-stromal paracrine pathway, involving not only PCa cells but also stromal cells. Interestingly, the use of this immune-compromised NSG tumor model reveals a tumor-suppressive effect of COX-2 inhibitors, which appears to be mediated through tumor-stromal interactions, independent of the inflammatory effects triggered by PGE2 (111). The dissection of the PGE2-prolactin paracrine signaling axis between the tumor and stroma provides mechanistic insights into the pathways targeted by COX-2 inhibition, which is the most compelling and epidemiologically validated chemoprevention strategy for human PCa (78). Furthermore, the pro-tumorigenic properties of cancer-associated fibroblasts (CAFs), compared to normal prostate fibroblasts, have been a subject of research for decades (112–114). Joseph et al. have identified and validated the *in situ* localization of three smooth muscle subtypes (prostate smooth muscle, pericytes, and vascular smooth muscle) and two novel fibroblast subtypes in the human prostate using scRNA-seq. In mice, both urethral fibroblasts (*Lgr5^+^*) and ductal fibroblasts (*Wnt2^+^*) contribute to a proximal Wnt/Tgfb signaling niche, which is absent in the human prostate. In contrast, human peri-epithelial fibroblasts express secreted WNT inhibitors such as SFRPs and DKK1, which may serve as a buffer against stromal WNT ligands by creating a localized signaling niche around individual prostate glands (115).

To sum up, CAF markers not only predict disease recurrence, metastasis, and survival in PCa patients but also mediate the response to specific therapies. Additionally, CAF signaling pathways are emerging as potential therapeutic targets, further underscoring the critical role of CAFs in the progression of PCa.

### Immune cells

The roles of immune cells in PCa have received growing attention in recent years (116, 117). Updated studies suggest that the initiation, progression, and castration resistance of PCa is influenced by interactions between tumor cells and the host immune system (88). Emerging evidence indicates that myeloid-derived cells, including tumor-associated macrophages (TAMs), tumor-associated neutrophils (TANs), myeloid-derived suppressor cells (MDSCs), and dendritic cells (DCs), are key innate immune cells that infiltrate the TME, contributing to tumor progression and treatment resistance of PCa patients (118, 119). In contrast, T cells (including both CD4+ and CD8+ subsets) and B cells are the predominant adaptive immune cells that infiltrate the prostate gland. Notably, PCa-infiltrating CD4+ T cells predominantly adopt T helper (Th) 17 and regulatory T (Treg) phenotypes, rather than the Th2 phenotype (120). CD8+ T cells exhibit dysfunction in both humans and transgenic mouse models of PCa (121, 122). In addition to these findings, the application of scRNA-seq to profile the diverse infiltrating immune cell populations in PCa offers valuable insights into cancer progression. The identification of novel TAM and T cell subpopulations through scRNA-seq will be discussed in the following paragraphs.

Among immune cells, TAMs play a pivotal role in regulating tumorigenesis (123, 124). A significant increase in TAMs, particularly M2-type TAMs, has been observed in metastatic PCa patients, suggesting that these cells contribute to tumor metastasis (125, 126). The development of scRNA-seq technology and advanced data analysis methods has made it possible to determine the molecular characteristics of various immune cell populations within the TME, providing a novel approach to identifying functional biomarkers (127, 128). Zhang and colleagues have investigated the roles of macrophage-related genes in molecular stratification, prognosis, TME, and immunotherapeutic response in PCa using public scRNA-seq databases. They have identified the NCF4 gene as the central gene in the macrophage-related risk signature, enhancing our understanding of macrophage infiltration in PCa and potentially offering new strategies for personalized treatment (129). Guan et al. have re-analyzed scRNA-seq data from the GEO database (GSE176031) and identified PPIF in TAMs as a potential biomarker and therapeutic target within the PCa microenvironment (130). Siefert et al. have demonstrated that TAMs express a combination of M1 and M2 markers, with TNF-α and NF-κB pathways being two major sources of variation between TAM subtypes. They have identified five distinct macrophage clusters, suggesting that expanding the patient sample size and increasing cell sequencing depth may improve our understanding of the role of TAMs in PCa (131). Li et al. have identified ATF3 as a significant macrophage-related risk signature that may be associated with docetaxel and cabazitaxel resistance in PCa cells, as well as with T stage and the Gleason score (132). Tuong et al. have created a single-cell transcriptomic map of the human prostate immune landscape in healthy conditions and demonstrated how it is disrupted in cancer. They have identified a prostate-specific macrophage population that helps maintain tissue zinc levels and is associated with improved cancer outcomes. Notably, the enrichment of the prostate-specific zinc transporter-expressing macrophage population (MAC-MT) signature in PCa biopsies is associated with improved disease-free survival, indicating potential antitumor benefits (133).

In addition to TAMs, T cells play a crucial role in the TME of PCa and serve as important indicators of immune activity (134–136). The scRNA-seq study by Chen et al. have revealed a novel subset of T cells with elevated levels of KLK3 in PCa tissue samples, a gene that encodes PSA. Profiling of a primary tumor and two matched lymph nodes provides evidence that ectopic expression of KLK3 is associated with micrometastases. In addition to KLK3, they have also detected PSA in extracellular vesicles (EVs) and in EV-co-cultured T cells (137). Similarly, Peng et al. have utilized scRNA-seq on clinical patient samples to identify the membrane receptor EP4 as a specific target for PCa immunotherapy. They have demonstrated that YY001 inhibits prostate tumor growth by regulating the immune microenvironment and strongly synergized with anti-programmed cell death protein (PD)-1 antibodies, converting completely unresponsive PCa into responsive cancers, resulting in significant tumor regression, long-term survival, and lasting immunological memory. These findings suggest that targeting EP4 with novel antagonists could serve as a therapeutic strategy for treating advanced PCa and enable effective combinatorial immunotherapy (138).

Briefly, the application of scRNA-seq has helped uncover the role of immune cells in PCa progression, particularly through the identification of prostate-specific macrophage and T cell subpopulations. Identifying new molecular targets enhances clinical outcomes and potentially prevent tumor recurrence in patients with PCa.

## Causal relationships of current findings in benign prostatic hyperplasia and prostate cancer via single-cell RNA sequencing

Emerging evidence from scRNA-seq is reshaping our understanding of the relationship between BPH and PCa. While historically treated as separate entities (139, 140),scRNA-seq atlases now reveal that these diseases are linked by a common ecosystem of pathogenic cell states driving prostatic proliferation. This section synthesizes how scRNA-seq uncovers convergent mechanisms in epithelial plasticity, stromal activation, and immune dysregulation that underlie both conditions. Zhang et al. provide evidence of a significant bidirectional causal relationship between genetically predicted BPH and an increased risk of PCa, demonstrating that the risk of PCa significantly rises with genetically predicted BPH (141). This finding aligns with the results of several researchers, who identified G-protein-coupled receptor-associated sorting protein 1, epigenetic DNA modifications, vitamin C, and the CYLD, JAK2, and TLR4 genotypes as important links between BPH and PCa (142–144). Based on these characteristics, this section primarily discusses the similarities and differences in current findings on BPH and PCa through scRNA-seq, with a focus on co-expressed biomarkers that may be highly valuable in guiding clinical practice.

In the 1960s, McNeal proposed the theory of prostate zones, dividing the prostate into three main regions: the transition zone (TZ), the CZ, and the peripheral zone (PZ) (145). It is widely acknowledged that prostatitis and PCa primarily occur in the PZ, BPH primarily occurs in the TZ, and the CZ is rarely involved in cancer or hyperplasia (146–148). To date, molecular differences in cell subpopulations across the three prostate zones remain poorly understood. The current scRNA-seq literature summarized in this review does not include spatial information across the prostate zones. Therefore, understanding the pathological changes of the prostate from the perspective of its compartmentalization through scRNA-seq is urgently needed.

Additionally, BPH and PCa share characteristics such as hormone-dependent growth and their response to antiandrogen therapy (149, 150). Androgen and its receptor play a crucial role in the physiological processes and pathological changes of the prostate (151, 152). Moreover, estrogen can influence prostatic carcinogenesis and neoplastic progression through an ER-β-mediated process in human prostate tissue (153–155). Published scRNA-seq studies have revealed that AR-expressed epithelial cells are involved in both BPH and PCa, through distinct mechanisms. Therefore, hormones may serve as key links between BPH and PCa. In the context of chronic prostate diseases, androgen deprivation therapy and 5-α-reductase (5AR) inhibition are commonly used in the treatment of men with advanced or metastatic PCa and BPH, respectively.

Meanwhile, risk factors such as prostate inflammation and metabolic disruption play key roles in the development of both diseases (156–158). The link between the immune system and the microenvironment of PCa is characterized by dynamic interplay, where immune cells have both inhibitory and promotive effects on cancer growth (159, 160). Previous scRNA-seq studies have identified several cell subpopulations expressing inflammation- and metabolism-associated markers, which contribute to the initiation and progression of both BPH and PCa. Su et al. have identified CHRDL1, NEFH, TAGLN, and SYNM as novel diagnostic biomarkers for BPH and PCa, which aid in early diagnosis, treatment, and assessment of immunotherapeutic responses. These biomarkers may have significant value in guiding clinical practice (161). However, only a few biomarkers differentiating between BPH and PCa have been identified, including proteins involved in MAP Kinase pathways and lipids containing oleic acid. These markers may offer a pathway to increased specificity (162). McNally et al. have reviewed hundreds of potential biomarkers in urine, serum, tissue, and semen, proposed as useful targets for distinguishing between PCa and BPH patients (163).

**Tables 1**, **2** together provide a comprehensive summary of current findings in BPH and PCa through scRNA-seq, as illustrated in **Figure 1** as well. Detailed information from each study reveals potential links. In terms of molecular mechanisms, BPH and PCa share androgen-dependent growth, chronic inflammation and specific microenvironmental changes that can be dissected by scRNA-seq. Recognizing the bidirectional relationship between BPH and PCa, men diagnosed with BPH could benefit from more stringent PCa screening protocols. In addition, translational potential of these findings expands the practical value of this article (**Figure 2**). Therefore, focusing on the relationship between BPH and PCa, either to connect or differentiate them, is important. scRNA-seq gives compelling evidence that BPH and PCa are not merely adjacent diseases but are driven by convergent cellular and molecular pathways.

**Figure 1:**
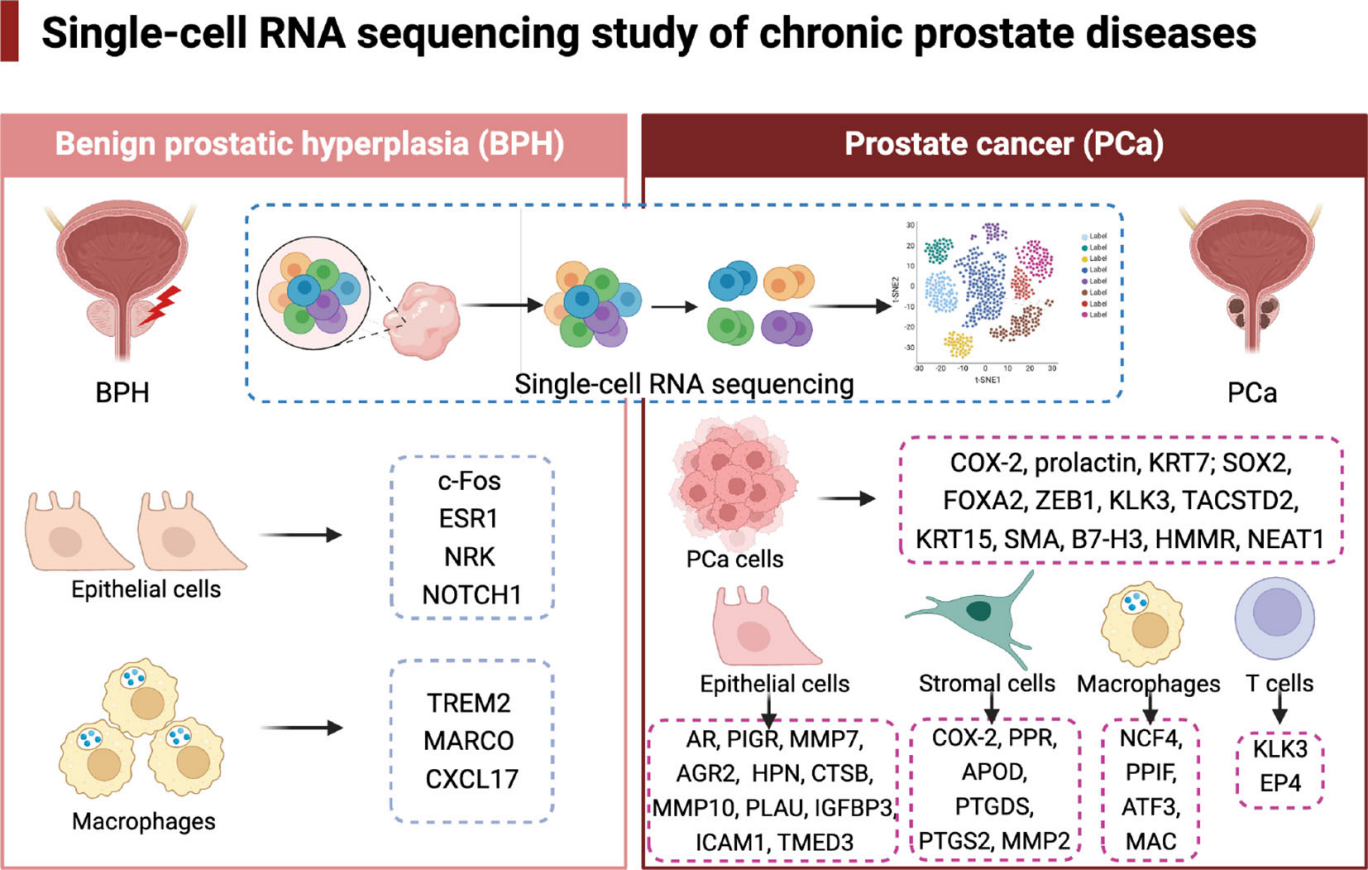
A summary of newly identified cell subpopulations in benign prostatic hyperplasia and prostate cancer through scRNA-seq. Depicted are specific epithelial cell, stromal cell, immune cell subpopulations with novel markers identified in BPH and PCa via scRNA-seq. A higher focus on PCa is paid compared to BPH.

**Figure 2:**
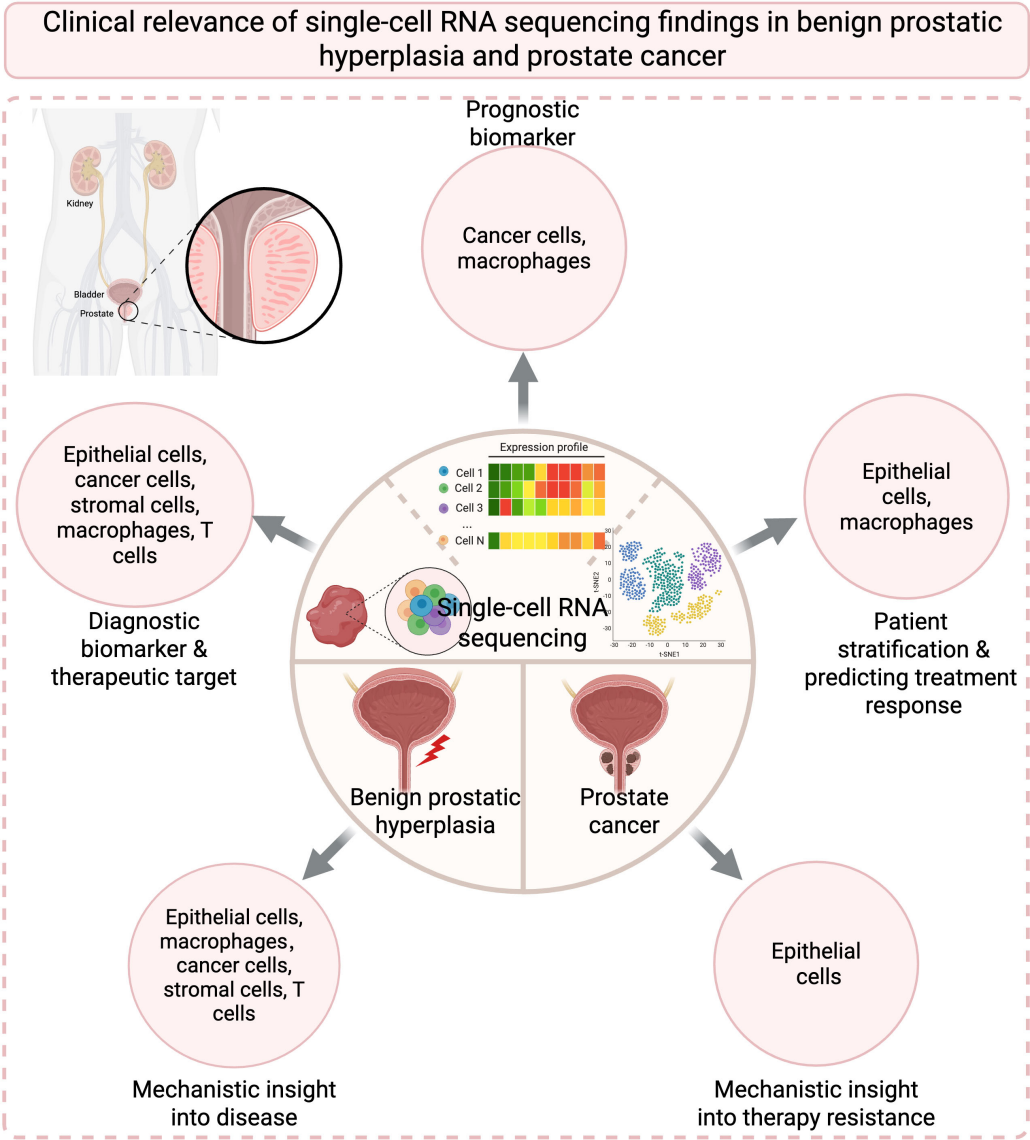
Clinical relevance of scRNA-seq findings in benign prostatic hyperplasia and prostate cancer. Depicted are cell subpopulations involved in five clinical relevant directions: diagnostic biomarker & therapeutic target, mechanistic insight into disease, mechanistic insight into therapy resistance, patient stratification & predicting treatment response, and prognostic biomarker.

## Concluding remarks and future perspective

The application of scRNA-seq to human and/or mouse prostate tissue marks a significant milestone in prostate research, demonstrating the technology’s ability to identify previously unrecognized subtypes. This review aims to highlight recent findings in BPH and PCa uncovered through scRNA-seq mainly through examining BPH and PCa progression from a single-cell perspective and summarizing specific marker-expressed cell subpopulations in two states. By drawing insights from representative, recently published articles, we explore the impact of this emerging technology on reinterpreting previous research and shaping the future direction of BPH and PCa studies. It is worth noting that early differentiation between BPH and PCa is crucial to prevent unnecessary and invasive tests in individuals. PSA testing alone cannot differentiate between BPH and PCa; thus, new biomarkers are essential for improving risk stratification at this stage. Further research using scRNA-seq is urgently needed to address these limitations including technical artifacts, biological interpretation challenges, and barriers to clinical translation, and explore the biological functions of newly discovered prostate-specific cell subpopulations.

The application of scRNA-seq is poised to fundamentally reshape the clinical management of BPH and PCa. A key translational output will be in biomarker development. Specific cell markers, such as those identifying a unique luminal progenitor population in BPH or a distinct subpopulation of epithelial cells with a partial EMT signature in PCa, hold immense diagnostic and prognostic potential. Furthermore, the characterization of specific immune cell subsets, like a pro-fibrotic macrophage population in BPH or a T-cell exhaustion signature in the PCa TME, could serve as powerful indicators for predicting disease progression and, crucially, for forecasting patient response to emerging immunotherapies. Validating these molecular features in liquid biopsies or targeted tissue assays will be the critical next step toward their clinical adoption.

Beyond diagnostics, scRNA-seq data provide an unprecedented roadmap for therapeutic target exploration and personalized medicine. The identification of pathogenic cell subpopulations, such as NRG1+ stromal fibroblasts in BPH or treatment-resistant basal cells in PCa, reveals novel, cell-specific drug targets. Intervention strategies could include monoclonal antibodies against surface markers unique to these cells or small molecule inhibitors targeting their critical survival pathways, such as aberrant Wnt or Hedgehog signaling. Ultimately, the integration of scRNA-seq profiles from large patient cohorts enables a move beyond traditional histology to achieve refined molecular subtyping. Clinicians may stratify BPH patients into “inflammatory,” “fibrotic,” or “epithelial-proliferative” subtypes, and PCa patients based on their dominant TME, thereby matching individuals with the most effective targeted therapies, immunotherapies, or combination treatments from the outset, and ultimately bridging the gap between high-resolution basic science discovery and tangible advances in precision medicine.
